# Protein hydrolysate from potato confers hepatic-protection in hamsters against high fat diet induced apoptosis and fibrosis by suppressing Caspase-3 and MMP2/9 and by enhancing Akt-survival pathway

**DOI:** 10.1186/s12906-019-2700-8

**Published:** 2019-10-25

**Authors:** Shibu Marthandam Asokan, Tsu-Han Hung, Zong-Yan Li, Wen-Dee Chiang, Wan-Teng Lin

**Affiliations:** 1 Department for Management of Science and Technology Development, Ton Duc Thang University, Ho Chi Minh City, Vietnam; 2 Faculty of Applied Sciences, Ton Duc Thang University, Ho Chi Minh City, Vietnam; 3 Department of Hospitality Management, College of Agriculture, Tunghai University, No.1727, Sec4, Taiwan Boulevard, Xitun, District, Taichung, 40704 Taiwan, Republic of China; 4 Department of Food Science, College of Agriculture, Tunghai University, Taichung, Taiwan

**Keywords:** APPH, Hepato-protection, Liver fibrosis, Liver apoptosis, High fat diet

## Abstract

**Background:**

A potato protein hydrolysate, APPH is a potential anti-obesity diet ingredient. Since, obesity leads to deterioration of liver function and associated liver diseases, in this study the effect of APPH on high fat diet (HFD) associated liver damages was investigated.

**Methods:**

Six week old male hamsters were randomly separated to six groups (*n* = 8) as control, HFD (HFD fed obese), L-APPH (HFD + 15 mg/kg/day of APPH), M-APPH (HFD + 30 mg/kg/day), H-APPH (HFD + 75 mg/kg/day of APPH) and PB (HFD + 500 mg/kg/day of probucol). HFD fed hamsters were administered with APPH 50 days through oral gavage. The animals were euthanized and the number of apoptotic nuclei in liver tissue was determined by TUNEL staining and the extent of interstitial fibrosis was determined by Masson’s trichrome staining. Modulation in the molecular events associated with apoptosis and fibrosis were elucidated from the western blotting analysis of the total protein extracts.

**Results:**

Hamsters fed with high fat diet showed symptoms of liver damage as measured from serum markers like alanine aminotransferase and aspartate aminotransferase levels. However a 50 day long supplementation of APPH effectively ameliorated the effects of HFD. HFD also modulated the expression of survival and apoptosis proteins in the hamster liver. Further the HFD groups showed elevated levels of fibrosis markers in liver. The increase in fibrosis and apoptosis was correlated with the increase in the levels of phosphorylated extracellular signal-regulated kinases (pERK1/2) revealing a potential role of ERK in the HFD mediated liver damage. However APPH treatment reduced the effect of HFD on the apoptosis and fibrosis markers considerably and provided hepato-protection.

**Conclusion:**

APPH can therefore be considered as an efficient therapeutic agent to ameliorate high fat diet related liver damages.

## Background

Obesity is a serious public health issue associated with chronic diseases of concern, such as type-II diabetes, cardiovascular disease, insulin resistance, fatty liver diseases, stroke, arthritis and asthma. The prevalence of obesity is on the rise in both developed and developing countries, where obesity at a young age is becoming a common phenomenon. According to WHO estimates the global prevalence of overweight adults is more than 1 billion and about 300 million of them are obese [1–5]. A high fat diet (HFD) containing high levels of lipids is a major causative factor for hepatic complications like hypercholesterolemia, steatohepatitis, inflammation, apoptosis and fibrosis [6–10]. However the molecular mechanisms behind HFD associated pathogenicity remain unclear and therefore an efficient counteracting therapeutic strategy is not yet available.

The liver plays an essential role in maintaining the lipid metabolism by controlling lipogenesis, lipolysis, gluconeogenesis, and glycolysis. Liver controls more than 10,000 biochemical reaction at a given time which helps in normal metabolic homeostasis and storage of carbohydrates, lipids, vitamins and minerals [11, 12]. Obesity is one of the most common conditions associated with liver disorders such as liver steatosis, nonalcoholic fatty liver disease and subsequent advancement to steatohepatitis. HFD intake significantly alters the molecular events and function of liver that is reflected with considerable modulation in the functional markers of liver [11].

Several studies involving obese humans and diet induced animal models have extensively reported on various adverse effects of HFD [13–17]. The Golden Syrian hamster a widely used animal model for lipoprotein metabolism and they are highly prone to obesity and associated disorders [18]. On a high fat diet, hamsters show significant body weight gain from the fourth week of treatment and continue to gain exponential growth at least up to the 12th week [18]. APPH is an alcalase hydrolysate of potato protein fraction possessing lipolysis-stimulating with efficient anti-obesity potential [19]. Nevertheless, the effect of APPH on high fat diet (HFD) induced hepatic apoptosis and fibrosis is uncertain.

While rats and mice models show resistance to develop hyperglycemia Golden Syrian hamsters are prone to obesity [20]. On a cholesterol-rich diet hamsters easily develop hypercholesterolemia and hypertriglyceridemia moreover, HFD easily induces obesity and fatty liver disease in hamsters [18, 21]. In order to study the hepato-protective effects of APPH, male Golden Syrian hamsters were fed with HFD for a period of 80 days and were found to exhibit the manifestations of hepatic damage. However, HFD fed hamsters administered with different doses of APPH for 50 days showed a considerable betterment from the symptoms. The ALT (alanine aminotransferase) and AST (aspartate aminotransferase) levels were found to be elevated in obese hamsters however APPH administration considerably reduced the serum levels of ALT and AST. The proteins involved in apoptosis and fibrosis were also seen to be significantly reduced in liver tissues of APPH administered obese hamsters. Our results reveal that prolonged APPH intake may attenuate HFD induced hepatic apoptosis and fibrosis in hamsters. Administration of APPH can be therefore considered as a potential therapeutic agent to ameliorate HFD related liver damages.

## Methods

### APPH preparation

Preparation, purification and characterization of APPH were performed as reported previously. The composition and characteristics of APPH were also verified to be consistent with that reported previously [22]. Briefly, potato protein (Han-Sient Corporation, Taipei, Taiwan) and alcalse enzyme were mixed (25:10 ratio) to obtain a protein hydrolysate with 81% proteins. The APPH was characterised by reverse phase HPLC and by MS/MS/TIC as mentioned in previous report [22].

### Animal experiments

This study was conducted following the IACUC-100-12 protocol and approved by the IACUC ethics committee. 1 week prior to the experiments, the hamsters (6 weeks old) were allowed to adapt to the experimental condition by housing them in a room maintained at 24 ± 2 °C and 55 ± 10% humidity with a 12 h light cycle. During adaptation all the animals were provided with a standard laboratory diet purchased from PMI Nutrition International, Brentwood, MO, USA and reverse osmosis treated water was provided ad libitum. After adaptation, the hamsters were randomly divided into six groups (*n* = 8): Control group hamsters were fed with standard chow, HFD group were fed HFD (containing 60% of energy as fat), HFD with low dose (15 mg/kg/day) APPH treatment, HFD with moderate (45 mg/kg/day) APPH treatment, HFD with high (75 mg/kg/day) APPH treatment and HFD with probucol (500 mg/kg/day). HFD was provided for a total of 80 days and oral APPH administration began after 30 days of HFD feeding and was provided for the next 50 days. Equal amounts of PBS were administered to the control and HFD group as a sedentary control. After 50 days of treatment the hamsters were anesthetized with overdose of isoflurane vapors using a calibrated vaporizer (5% isoflurane) and the unconscious animals were decapitated.

#### Determination of serum markers

For serum analysis the blood samples were drawn from the hamsters and centrifuged at 2000 rpm for 10 min and ALT and AST levels were measured by using commercially available assay kits (Abcam, Cambridge, United Kingdom).

#### Protein extraction from tissue samples

Liver tissue sections were homogenized in lysis buffer (100 mg/mL) containing Tris, EDTA, 2-mercaptoethanol, 10% glycerol, protease inhibitor and phosphatase inhibitor (pH = 7.4). The supernatants containing soluble proteins were collected by centrifuging the homogenates at 12,000 *g* for 40 min.

#### Western blotting analysis

Protein concentration of the samples was determined using Lowry’s protein assay method. Proteins were separated by Sodium dodecyl sulfate−polyacrylamide gel electrophoresis (SDS-PAGE) and were subsequently transferred to PVDF (GE Healthcare Life Sciences, Pittsburgh, PA, USA) membranes. The membranes were blocked using 3% bovine serum albumin (BSA) in TBS buffer and then hybridized with primary antibodies (Santa Cruz Biotechnology, Santa Cruz, CA, USA). After proper washing with TBS buffer the membranes were hybridized with horseradish peroxidase-labelled secondary antibodies and the blots were visualized with ECL in a Fujifilm LAS-3000 (GE Healthcare Life Sciences) chemiluminescence detection system.

### Tissue staining

Masson’s trichrome staining to determine hepatic fibrosis and Terminal Deoxynucleotide Transferase-mediated dUTP Nick End Labeling (TUNEL) assay to determine apoptosis were performed on paraffin embedded tissues as mentioned previously [22]. Briefly, tissue slides were dewaxed and rehydrated by decreasing concentration of alcohol and stained with Masson’s trichrome dye. For TUNEL assay the sections were treated with proteinase K followed by permeablization solution and then incubated in TUNEL reagent (Roche Applied Science, Indianapolis, IN, USA) for 60 min at RT. The sections were washed in PBS at least twice between each successive step. The sections were appropriately photographed with Olympus DP74 camera (Olympus, Tokyo, Japan) fitted to a microscope (BX53, Olympus). TUNEL sections were photographed under fluorescence to detect the TUNEL positive nuclei in green and the DAPI counter stained nuclei in blue.

### Statistical analysis

The results presented are the means ± SD obtained from three independent experiments. Statistical analysis was performed using ANOVA one-way analysis of variants.

## Results

### APPH administration supresses HFD induced apoptosis and fibrosis

**Fig. 1:**
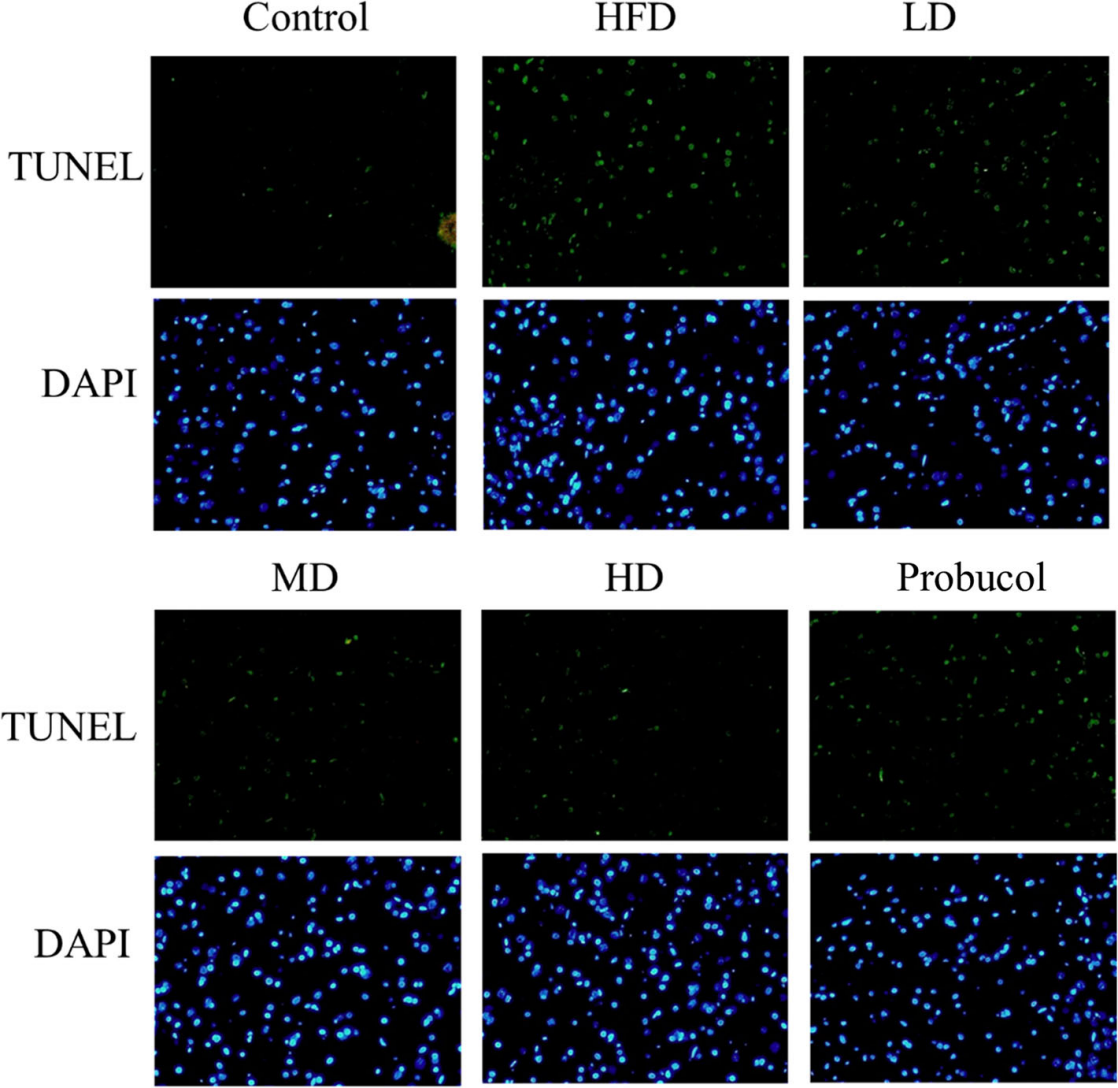
Effect of APPH on hepatic apoptosis. TUNEL assay results show apoptotic nuclei (green) among the total nuclei (blue) in Control hamsters, HFD fed hamsters (HFD), HFD fed hamsters treated with low dose of APPH (L-APPH), HFD fed hamsters treated with moderate dose of APPH (M-APPH), HFD fed hamsters treated with high dose of APPH (H-APPH) and HFD fed hamsters treated with probucol

**Fig. 2:**
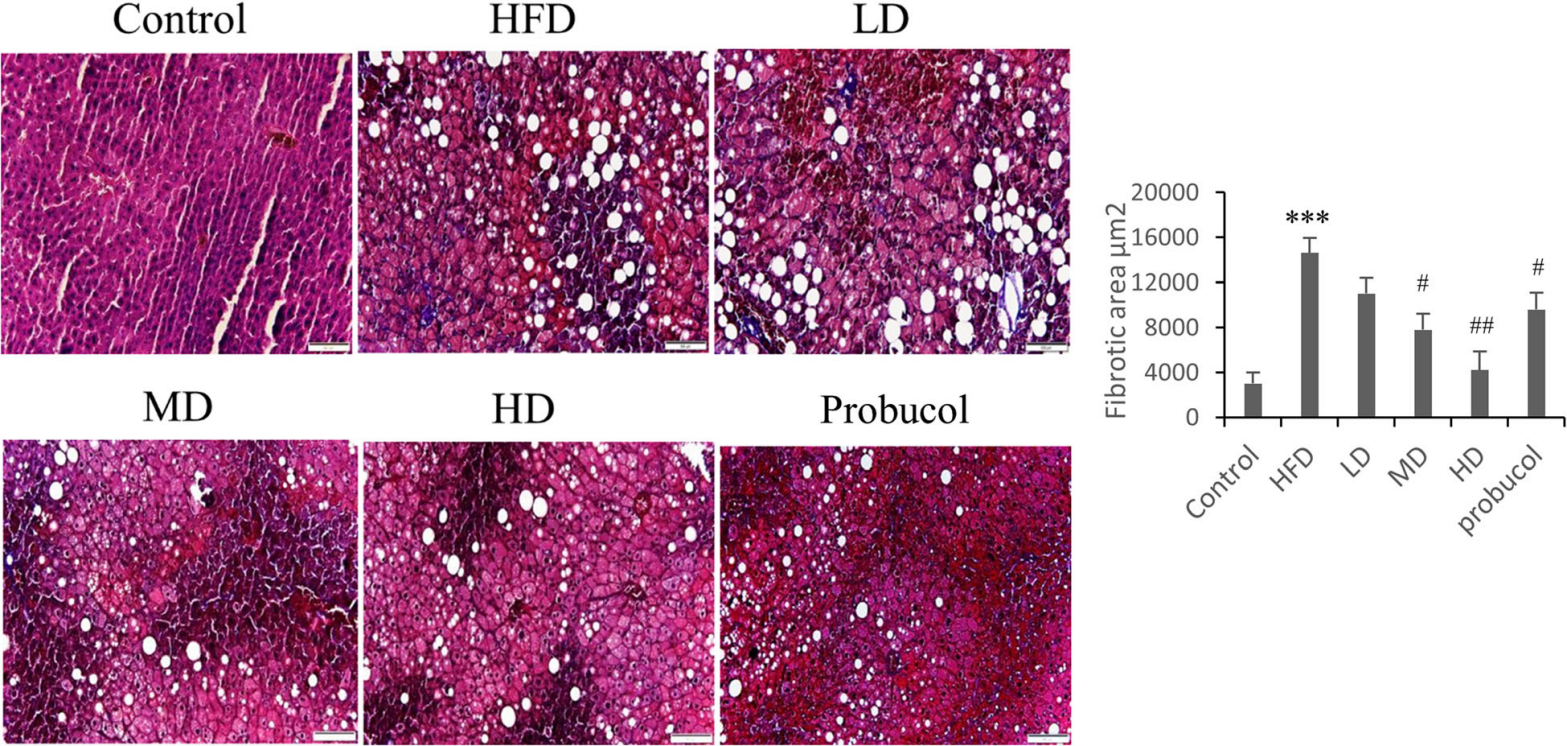
Effect of APPH on hepatic fibrosis: Masson’s trichrome staining show the levels of collagen accumulation in HFD and the APPH treated hamsters in Control hamsters, HFD fed hamsters (HFD), HFD fed hamsters treated with low dose of APPH (L-APPH), HFD fed hamsters treated with moderate dose of APPH (M-APPH), HFD fed hamsters treated with high dose of APPH (H-APPH) and HFD fed hamsters treated with probucol. *n* = 3, ^***^ *p* < 0.001 when compared with the Control group; ^#^ *p* < 0.05 and ^##^ *p* < 0.01 when compared with HFD group

TUNEL staining on liver tissue sections showed increase in the number of TUNEL positive cells in HFD rat groups. However, administration of low, moderate and high doses of APPH effectively supressed apoptosis as seen from the reduction in the number of apoptotic nuclei stained in green (Fig. 1). Effect of APPH administration on HFD induced apoptosis was also seen to be superior to that of probucol. Masson’s trichrome staining of liver tissue sections showed that HFD in hamsters triggered hepatic fibrosis which was substantially suppressed in hamsters treated with APPH as seen from the reduction their collagen accumulation (Fig. 2).

### Effect of APPH on the levels of the markers of liver injury

The levels of the serum aminotransferases, including ALT and AST in the HFD group increased significantly when compared to the control groups. However, the levels remained low in the APPH administered hamsters groups indicating a reduction in the HFD induced liver damage (Table 1).

**Table 1:** Changes in serum markers representing liver damage

Markers(mg/dL)	Control *n* = 8	HFD *n* = 8	LD *n* = 8	MD *n* = 8	HD *n* = 8	PB *n* = 8
AST	221 ± 18	267 ± 30	174 ± 22^###^	177 ± 23^###^	156 ± 20^###^	231 ± 34
ALT	84 ± 3	426 ± 67^***^	291 ± 43^###^	271 ± 52^###^	314 ± 26^###^	310 ± 46^###^

*HFD* High-fat-diet, *LD* Low-dose APPH (15 mg/kg/day), *MD* Moderate dose APPH (45 mg/kg/day), *HD* High-dose APPH (75 mg/kg/day), *PB* probucol (500 mg/kg/day). ^***^ *p* < 0.001 when compared with the Control group; ^###^ *p* < 0.001 when compared with HFD group

### APPH administration attenuates hepatic apoptosis and enhance survival related proteins

**Fig. 3:**
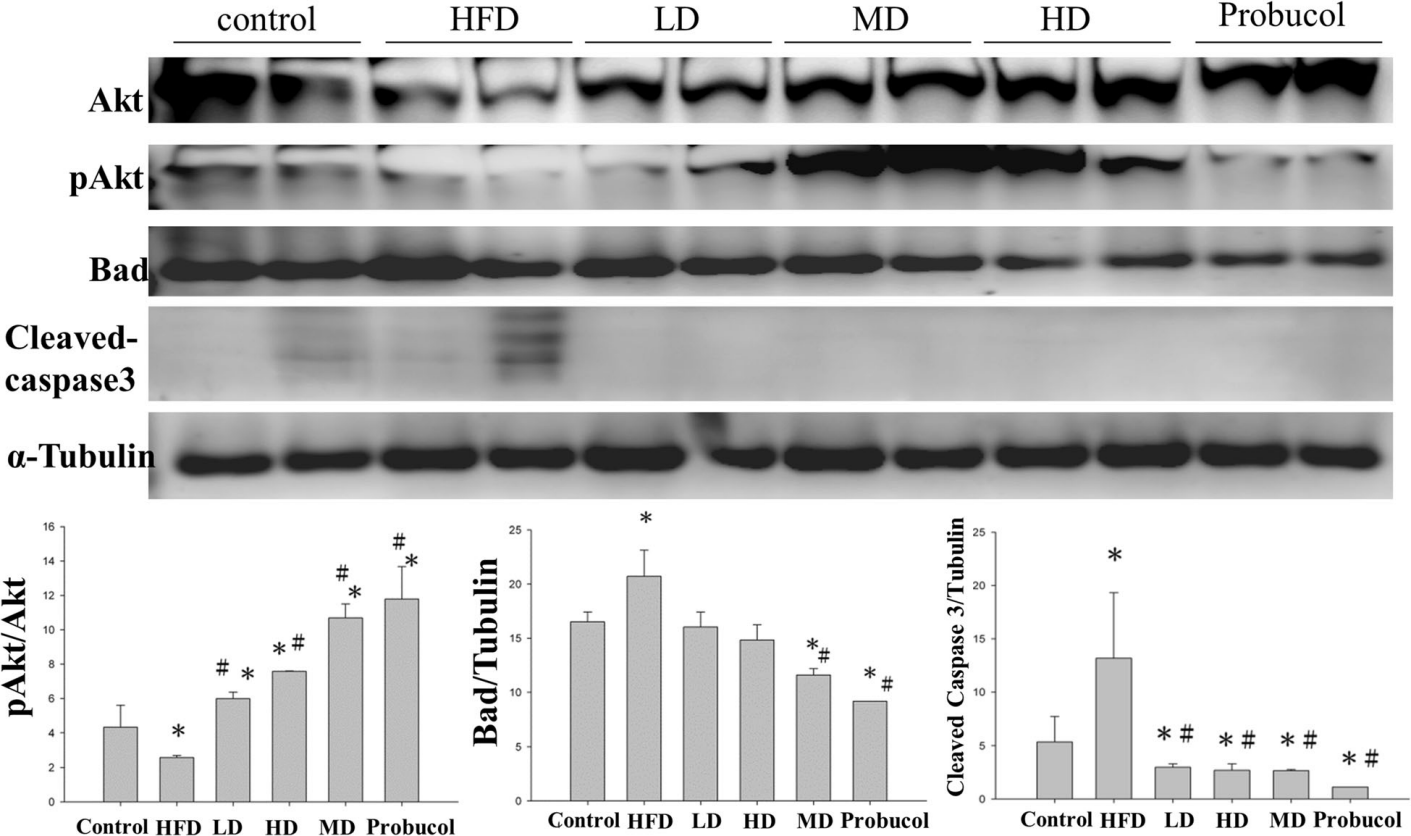
Apoptosis and survival Protein expression analysis by western blotting. Levels of apoptosis and survival related proteins in the liver sections of Control, HFD fed hamsters (HFD), HFD fed hamsters treated with low dose of APPH (L-APPH), HFD fed hamsters treated with moderate dose of APPH (M-APPH), HFD fed hamsters treated with high dose of APPH (H-APPH) and HFD fed hamsters treated with probucol. *n* = 5, ^*^ *p* < 0.05 when compared with the Control group; ^#^ *p* < 0.05 when compared with HFD group

Analysis of protein expression by western blotting showed that HFD feeding in hamsters down-regulated the survival proteins Akt and up-regulated the apoptotic proteins such as cleaved caspase 3 and Bad. Hamsters that were provided with low, moderate or high levels of APPH showed suppressed levels of Bad and caspase 3 (Fig. 3).

### APPH administration regulates MMP2 and MMP9

**Fig. 4:**
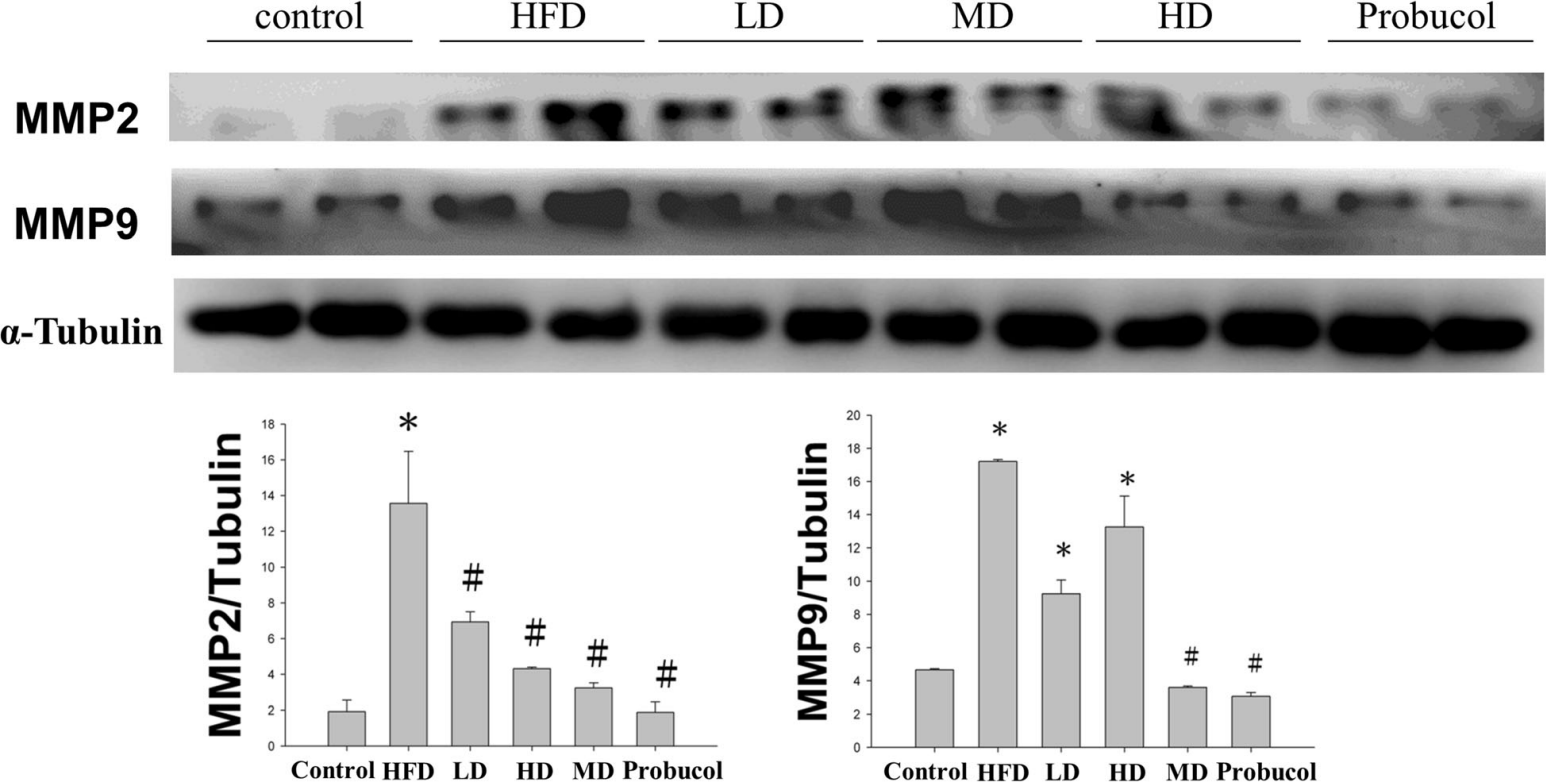
Expression analysis of Fibrosis related proteins by western blotting. Levels of apoptosis and survival related proteins in the liver sections of Control, HFD fed hamsters (HFD), HFD fed hamsters treated with low dose of APPH (L-APPH), HFD fed hamsters treated with moderate dose of APPH (M-APPH), HFD fed hamsters treated with high dose of APPH (H-APPH) and HFD fed hamsters treated with probucol. . *n* = 5, ^*^ *p* < 0.05 when compared with the Control group; ^#^ *p* < 0.05 when compared with HFD group

Hamsters that fed on HFD showed high levels of MMP2 and MMP9 in the liver, increase in these MMPs in liver generally correlates with liver fibrosis. However, the levels were significantly reduced when treated with APPH revealing the protective effects developed in the treatment groups against hepatic fibrosis (Fig. 4).

### Effect of APPH on MAPK kinase expression

**Fig. 5:**
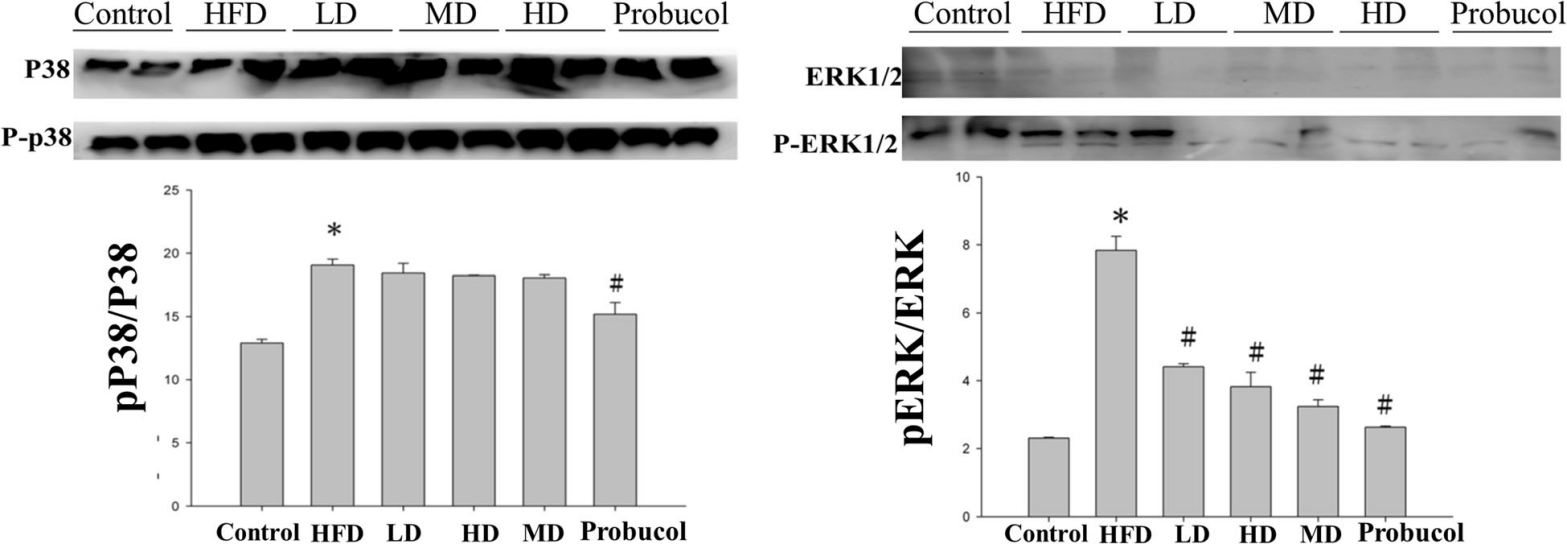
Expression analysis of MAPK proteins by western blotting. Levels of apoptosis and survival related proteins in the liver sections of Control, HFD fed hamsters (HFD), HFD fed hamsters treated with low dose of APPH (L-APPH), HFD fed hamsters treated with moderate dose of APPH (M-APPH), HFD fed hamsters treated with high dose of APPH (H-APPH) and HFD fed hamsters treated with probucol. *n* = 5, ^*^ *p* < 0.05 when compared with the Control group; ^#^ *p* < 0.05 when compared with HFD group

HFD feeding in hamsters resulted in the increase of phosphorylated MAPKs such pP38 and pERK. The APPH significantly suppressed the expression of pERK and did influence the levels of pP38 (Fig. 5). However probucol showed a moderate effect in reducing the effect of HFD in P38 signaling indicating that probucol acts by influencing multiple pathways.

## Discussion

Obesity is generally associated with imbalance in energy intake and expenditure and it inflicts substantial burden on health as it is linked with the pathogenesis of various common diseases like type-II diabetes, cardiovascular disease and NAFLD [23–25].

The liver is a vital organ for metabolism, detoxification and for regulating of immune responses. Therefore, liver is susceptible to injury following exposure to various stresses and in response to injury new extracellular matrix is synthesized as a process of healing. Regulation of ECM is governed by production and proteolysis and is crucial to maintain liver structural and functional homeostasis. Several factors such as the MMPs play a major role in maintain the balance between fibro genesis and fibro lysis and their dysregulation leads to hepatic fibrosis. Liver tissue MMPs play a crucial role in fibrogenesis. Among the known MMPs only a few are normally found in liver tissue. MMP-2 which is hardly detectable in rodents under normal conditions is expressed by hepatic stellate cells in response to liver injury. MMP-2 for being an autocrine factor in hepatic stellate cells for proliferation and migration enhances liver fibrosis when overexpressed [26].

Our previous study showed that APPH administration effectively suppresses HFD induced apoptosis in aging rats. However the hepatic collagen accumulation induced by HFD is not very prominent in aging rats [22]. In aging models the process of fibrolysis is often impaired and therefore results in excess accumulation of ECM factors and thereby results in hepatic fibrosis [27]. Therefore in order to authenticate the fibrolysis effects induced by APPH, it is essential to evaluate their effects in young rats. The results in the present study shows that APPH administration results in suppression of MMP-2 and MMP-9 and thereby suppress the fibrosis effects in a dose dependent manner as seen from Masson’s trichrome staining.

Serum aminotransferases are considered as important indicators of liver damage [28]. Usually the ALT activity in the liver is around 3000 times higher than that in the serum. However, during hepatic injury ALT is released in the serum causing increase serum levels of ALT [28]. While ALT is found in large quantities in the cytosol of hepatocyte, AST is present in substantial levels in a wide variety of tissues and is higher in kidney, heart, and skeletal muscle than in liver. Increase in AST activity indicates changes occurred in hepatocellular membrane potential, cellular necrosis and inflammation [29, 30]. Our data show that levels of both AST and ALT were significantly increased in the serum proteins of hamsters fed with HFD. The results indicate the HFD feeding deteriorated the liver function and affected the overall health of the animal. Meanwhile the APPH administration in hamsters reduced the levels of ALT and AST considerably and shows an enhanced liver function and general health of the HFD fed hamsters.

The members of the Bcl-2 family of proteins-Bcl-2 and Bad are well known markers of apoptosis; while Bcl-2 is an anti-apoptotic protein, Bad is pro-apoptotic [31]. Our results demonstrate that the markers of intrinsic and extrinsic apoptosis which were higher in HFD group hamsters were found to be regulated upon APPH administration. The APPH also suppressed the levels of Bcl-2 and up-regulated Bax protein levels in the liver tissue of HFD fed hamsters. Therefore, the results reveal the anti-apoptotic potential of APPH against HFD induced liver damages.

The PI3K/Akt signaling pathway is a crucial survival mechanism that counteracts against apoptotic events in most cells [32]. To determine if the beneficial effects of APPH on HFD induced hepatic apoptosis in hamsters involves PI3K/Akt mechanism, the levels of active (phosphorylated) form of Akt (p-Akt) and total Akt were analyzed.

Various evidences suggest that MAPK-signaling is involved in mitochondria-mediated intrinsic apoptosis [33]. In the present study, the level of phosphorylated p38 was significantly altered in HFD group hamsters however; APPH did not show any significant ameliorating effect. However extracellular signal-regulated kinase (ERK) which was highly expressed in the HFD fed hamsters were significantly regulated in the hamsters treated with APPH. This suggests a prominent role of ERK in HFD induced apoptosis. Moreover, APPH treatment completely restored Akt phosphorylation that was suppressed by HFD feeding. The results thus suggest that APPH administration associated anti-apoptotic effect is mediated through inactivation of ERK mechanism and activation of the PI3K/Akt signaling in a dose dependent manner.

Apoptosis and associated events prompt the migration of stellate cells to the region of apoptosis to engulf the apoptotic bodies in the liver. Hepatic stellates in the site of tissue damage involves in the deposition of extracellular matrix which is also associated with wound healing [34]. Dysregulation in the healing process results in scar formation which may be further subjected to progression of hepatic fibrosis. Increase in MMP level and activity is one of the characteristic events associated with such wound healing process. In a rat model with bile duct ligation induced hepatic fibrosis, MMP-2 and MMP-9 activities has been demonstrated to increase within 2 days post ligation therefore, MMP-2 and MMP-9 are considered to be suitable markers for the onset of liver fibrosis [34, 35]. The results show increase in hepatic MMP-2 and MMP-9 levels in hamsters in HFD group but the levels were found to be regulated in APPH administered hamster.

## Conclusion

In this study, the potential of APPH to overcome HFD induced apoptosis and fibrosis in the livers of HFD fed hamsters was verified with low, moderate and high doses APPH. APPH administration showed better effects against hepatic damages compared to probucol and therefore HFD triggered damages in the liver may be reversed with APPH administration.

## Data Availability

The datasets used and/or analyzed during the current study are available from the corresponding author on reasonable request.

## Abbreviations

ALT: Alanine Aminotransferase
APPH: Alcalase derived potato protein hydrolysate.
AST: Aspartate Aminotransferase
HFD: High Fat Diet
